# Does one size fit all? Developing an evaluation strategy to assess large language models for patient safety event report analysis

**DOI:** 10.1093/jamiaopen/ooae128

**Published:** 2024-11-09

**Authors:** Allan Fong, Katharine T Adams, Christian Boxley, Josanne A Revoir, Seth Krevat, Raj M Ratwani

**Affiliations:** Center for Biostatistics, Informatics, and Data Science, MedStar Health Research Institute, MedStar Health, Washington, DC 20008, United States; Center for Biostatistics, Informatics, and Data Science, MedStar Health Research Institute, MedStar Health, Washington, DC 20008, United States; Center for Biostatistics, Informatics, and Data Science, MedStar Health Research Institute, MedStar Health, Washington, DC 20008, United States; MedStar Institute for Quality and Safety—MedStar Health, Columbia, MD 21044, United States; Center for Biostatistics, Informatics, and Data Science, MedStar Health Research Institute, MedStar Health, Washington, DC 20008, United States; Medstar Georgetown University Hospital, Washington, DC 20007, United States; Georgetown University School of Medicine, Washington, DC 20007, United States; Georgetown University School of Medicine, Washington, DC 20007, United States; MedStar Health National Center for Human Factors in Healthcare—MedStar Health, Washington, DC 20008, United States

**Keywords:** patient safety, patient safety event reports, incident reports, perplexity, large language, models, clinical large language models

## Abstract

**Objective:**

Collecting and analyzing patient safety event (PSE) reports is a key component to the improvement of patient safety yet report analysis has been challenging. Large language models (LLMs) may support analysis; however, PSE reports tend to be a hybrid of clinical and general language.

**Materials and Methods:**

We propose a data-driven evaluation strategy to assess LLM fit for report analysis. We identify target tokens and sentences from PSE reports and use perplexity to evaluate four LLMs comprehension of the target sentence.

**Results:**

LLMs had statistically significantly different perplexity measures in six of seven event categories. Clinical models perform better with clinical narratives, often reported by nurses and physicians. General models perform better with colloquial language and communication themes.

**Discussion and Conclusion:**

For LLMs to support PSE report analysis there must be a good fit between the language model and the nature of the text in reports. A single LLM approach may not be the most useful strategy.

## Introduction

A key component to improving patient safety is the reporting of near misses, defined by the Agency for Healthcare Research and Quality (AHRQ) as an unsafe situation that is indistinguishable from a preventable adverse event except for the outcome, and adverse events (ie, instances where a patient was harmed), the analysis of these reports, and the development of interventions to address the factors contributing to these events.^1^ Event reporting is part of the World Health Organizations Global Patient Safety Action Plan and is encouraged by stakeholders such as the AHRQ and the Joint Commission.^1^^,^^2^ Most healthcare facilities in the United States have a software platform to collect patient safety event (PSE) reports from frontline healthcare workers.

PSE reports contain a free text description of the event and through analysis of this free text significant insights may be gleaned to inform improvement efforts. Many healthcare facilities have tens of thousands of reports to analyze and there are few tools to support analysis resulting in laborious efforts from patient safety analysts as they manually review reports.^3^ Recent research has also highlighted that across federal agencies and other stakeholders there are over 28 million safety reports, distinct from those collected by healthcare facilities, with few, if any, usable tools to derive insights from these reports.^4^

Recent advances in artificial intelligence, specifically large language models (LLMs), affords the opportunity to develop methods to support the more rapid and rigorous analysis of PSE reports. LLMs have the potential to transform PSE report analysis by reducing resources required to analyze reports and improving the insights that can be gleaned. Most biomedical and health related LLMs have been developed with a variety of publicly available clinical text, medical conversations, publications, and other healthcare related text.^5^ PSE reports differ from other clinical text, such as clinical documentation, since usually anyone (clinical or non-clinical) in a healthcare facility can submit (eg, nurses, pharmacists, transporters, environmental services staff, administrators, physicians, therapists) and the language and structure vary compared to clinical notes. PSE reports also tend to be a hybrid of clinical language and general language which may pose challenges for LLMs.^6^ Furthermore, PSE reports are considered protected data from a liability perspective and must be carefully managed. Thus, to fully leverage LLMs in PSE report analysis by healthcare systems and safety leaders, it is necessary to identify which LLMs might most accurately support PSE report text. Many LLM evaluation and benchmark methods involve the use of public collections of large, annotated datasets and diagnostics tests.^7–9^ While these publicly available datasets, metrics, and tests allow for easier comparisons of newer models, a simpler evaluation strategy that can be implemented with local data is needed to identify LLMs that can most accurately support the analysis of PSE report text. Thus, we sought to develop a lightweight method to assess the fit of different LLMs to PSE report analysis.

## Methods

### Development of a LLM evaluation strategy

**Figure 1.**
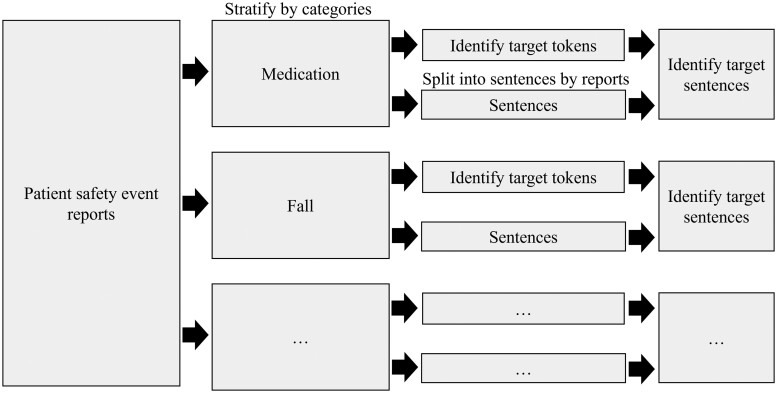
Pipeline for identifying target tokens and target sentences.

We propose a data-driven strategy informed by clinical experts to first identify a set of target tokens and target sentences from PSE reports, Figure 1. We then evaluate the target sentence perplexity difference between LLMs for each event category.^10^ The perplexity measure is chosen as it considers the log likelihood of each word in a given sentence as well as its general sequence and, as such, a lower perplexity suggests less uncertainty and a better fit. Perplexity is a metric that can be used for assessing how well a LLM predicts text. Our use of perplexity is intended to evaluate a LLM’s ability to generate real-world PSE text. Perplexity was chosen over other evaluation metrics such as ROUGE as the latter are typically used for more downstream summarization tasks.^11^ Since we chose an algorithmic approach to select target sentences starting with target tokens, the sentences may not necessarily be summaries. We chose this approach because we believe this strategy of starting with target tokens and then target sentences was more intuitive and we sought to develop this lightweight method to generally assess the “fit” of different LLMs to PSE reports. Although perplexity can be impacted by vocabulary size and limited coherence, this metric can be a useful way to evaluate how well different LLMs capture the concepts in the sentences for different event categories.^12^^,^^13^

### Data source

A total of 47 243 PSE reports from a mid-Atlantic healthcare system in the United States were sampled between January 1, 2022 to January 8, 2023. Reports belonged to 1 of 7 common event categories: diagnosis in treatment events (DX), medication and fluid related events (MED), falls (FALL), staff safety and security events (SFTY), patient identification and documentation events (PID), skin and tissue events (SKN), and professional conduct (PROF). These seven categories were chosen (out of a total of 26 categories) because they both represent the diversity of safety themes discussed in PSE reports and together represent over a majority, 56%, of all PSE reports. Specifically, medication and fluid related events account for 17% of the reports, falls account for 15%, skin and tissue events account for 7%, staff safety and security account for 6%, diagnosis in treatment account for 5%, and professional conduct and patient identification and documentation each account for 3%. This research was approved by the Institutional Review Board (IRB) at MedStar Health Research Institute (IRB ID: STUDY00002189).

### Identifying target tokens

We first consider each token, *t*, in the PSE vocabulary, *V*, for each event category in *N*. We calculate the conditional probability of a token, *t_i_*, occurring given the probability of category n_*j*_:

$$t_{j}=\left\{t_{i\in V}:\;p\left(t_{i}|n_{j}\right)>\sum_{k\in N,\;k\neq j}p(t_{i}|n_{k})\right\}$$

Tokens are then selected for a category, *n_j_*, when the conditional probability for *t_i_* given *n_j_* is greater than the sum of probabilities of *t_i_* conditioned for all other categories. This strategy is motivated by entropy comparisons as it provides a way to ensure tokens that are most likely assigned to a category will account for the frequency distribution of reports.^14^ We create a list of tokens for each event category that is then reviewed and further filtered by 2 clinical safety experts with over 15 years of experience for relevance and to determine final inclusion. The clinical safety experts prioritized tokens directly relevant to the safety event itself over those that were more indicative of general departmental policies and procedures for reporting PSEs. The selected tokens become the target tokens. Each event category has a different set of target tokens.

### Identifying target sentences

Next, we select a minimal set of sentences from PSE reports that can cover the list of target tokens for each category. A PSE report can have multiple sentences and we want to select sentences that come from different reports to avoid oversampling from the same report. To address this constraint, we define the sentence selection problem as a constraint satisfaction problem following a depth first search greedy approach.^15^ Our algorithm, below, initializes with an empty sentence set and an empty set of discovered reports. The algorithm first orders each sentence based on the tokens in W in each sentence. The sentence with the most tokens is chosen first. The sentence is valid if it is from a report that is not previously visited. If the sentence is valid, the report and sentence are added to the visited lists and the associated token(s) are removed from W. If the sentence is not valid, the algorithm will incrementally try each of the remaining sentences. If there are no valid sentences left, the algorithm will backtrack to traverse the search space for the next best option.

**ALGORITHM:** Selecting target sentences *1.  W ← List of unvisited tokens*

*2.  SENTS ← list of unvisited sentences with at least one token*

*3.  S = [] ← list of visited sentences*

*4.  R = [] ← list of visited reports*

*5.*

*6* ***.  while token in W:***

*7.   ORDERED_SENTS ← order SENTS by number of tokens from W in sentence*

*8.   s_r_[0] ← select top sentence from ORDERED_SENTS*

*9.   **if** r[0] is in R:*

*10.     ORDERED_SENTS.remove(sr[0])*

*11.     **while** r in R and ORDERED_SENTS is not empty:*

*12.      s_r_ ← try next sentence in ORDERED_SENTS*

*13.      **if** r in R: remove sentence from ORDERED_SENTS*

*14.     **if** ORDERED_SENTS is []:*

*15.      find backtracked tokens associated with r[0] and put back in W*

*16.      add sentences associated with backtracked tokens back into SENTS*

*17.      remove sentences from S*

*18.       R.remove(r[0])*

*19.   **else:***

*20.     S.add(s_r_)*

*21.     R.addI*

*22.     SENTS.remove(s_r_)*

*23.     W.remove(tokens in s_r_)*

### Comparing pretrained LLMs

Four LLMs, 2 general LLMs (GPT2 and Falcon-7B) and 2 medical LLMs (BioMedLM and MedAlpaca-7B), were chosen for comparison, detailed in Table 1. Two general LLMs (GPT2 and Falcon-7B) were trained on web data and have a larger vocabulary than the two models (BioMedLM and MedAlpaca) finetuned on medical data. GPT2 was trained on 40GB of English language web scraped data and has 124 million parameters and a vocabulary size of 50 257.^16^ Falcon-7B is a model trained on 1500 billion tokens from web data and has a vocabulary of 65 024.^17^ BioMedLM is a domain-specific model trained by Stanford Center for Research on Foundation Models and MosaicML on abstracts and papers from an online repository, The Pile.^18^ BioMedLM has a vocabulary size of 28 896 and is trained out to 300 billion tokens. MedAlpaca-7b is a 7 billion parameter model based on LLaMA and finetuned on 400k items of medical domain specific data the authors have referenced as Medical Meadow. It has a vocabulary size of 32 001.^19^ The BioMedLM uses a custom tokenizer trained on domain specific texts which allows for a more accurate encoding of information about medical terms. For instance, “cytotoxicity” is considered a single tokenizer by BioMedLM and split into 3 tokens with a standard GPT2 tokenizer. The other three models employ a base tokenizer that uses the Byte Pair Encoding algorithm.

**Table 1.** Comparison of LLM characteristics.

	GPT2	Falcon-7B	BioMedLM	MedAlpaca-7B
**Vocabulary domain**	General	General	Medical	Medical
**Base model**	—	—	GPT2	LLaMA
**Training data**	Web data, English language	Web data, English language	Medical abstracts and full papers	Medical Meadow
**LLM parameter size**	124 million	7 billion	2.7 billion	7 billion
**Vocabulary size**	50 257	65 024	28 896	32 001
**Tokenizer**	Base	Base	Custom	Base

The 2 general models were selected due to their overall performance and wide use, while the 2 medical domain specific models were chosen due to the varied medical data used during training. These 4 models are relatively lightweight compared to more powerful, larger alternatives due to the practicality of space requirements for local inference. The LLMs were implemented using the Huggingface evaluation perplexity metric function for each target sentences with batch sizes of 16 and positive starting token probabilities.^20^ The sequence length for BioMedLM, MedAlpaca, and Falcon7B were set to 2048 tokens, while sequence length for GPT2 was set to 1024 tokens. Analysis of variance was used to evaluate the target sentence perplexity difference between LLMs for each event category. Lastly, we reviewed individual target sentence perplexities with clinical safety experts and model running times.

## Results

A total of 1639 tokens were initially identified by their conditional probabilities and frequency distributions across event categories. Two clinical safety experts reviewed and removed tokens they considered not relevant to the associated event category. This resulted in the final 1409 target tokens and 600 target sentences used for the LLM perplexity evaluation, summarized in Table 2.

**Table 2.** Number of target tokens and target sentences across seven event categories: skin and tissue (SKN), medication and fluid (MED), staff safety and security (SFTY), fall (FALL), professional conduct (PROF), diagnosis in treatment (DX), and patient identification and documentation (PID). Average perplexity for each LLM by each event category.

	SKN	MED	SFTY	FALL	PROF	DX	PID
**# target tokens**	701	192	198	144	110	35	29
**# target sentences**	231	102	96	69	66	18	18
**GPT2**	375.1	299.6	297.8	246.7	142.3	315.7	303.5
**Falcon-7B**	114.7	81.1	147.7	97.5	73.7	**104.5**	141.6
**BioMedLM**	110.1	141.1	121.0	104.9	90.6	132.1	**138.9**
**MedAlpaca-7B**	**85.9**	**66.3**	**115.9**	**73.6**	**63.8**	105.6	180.0

The lowest average perplexity for each event category is bolded.

**Figure 2.**
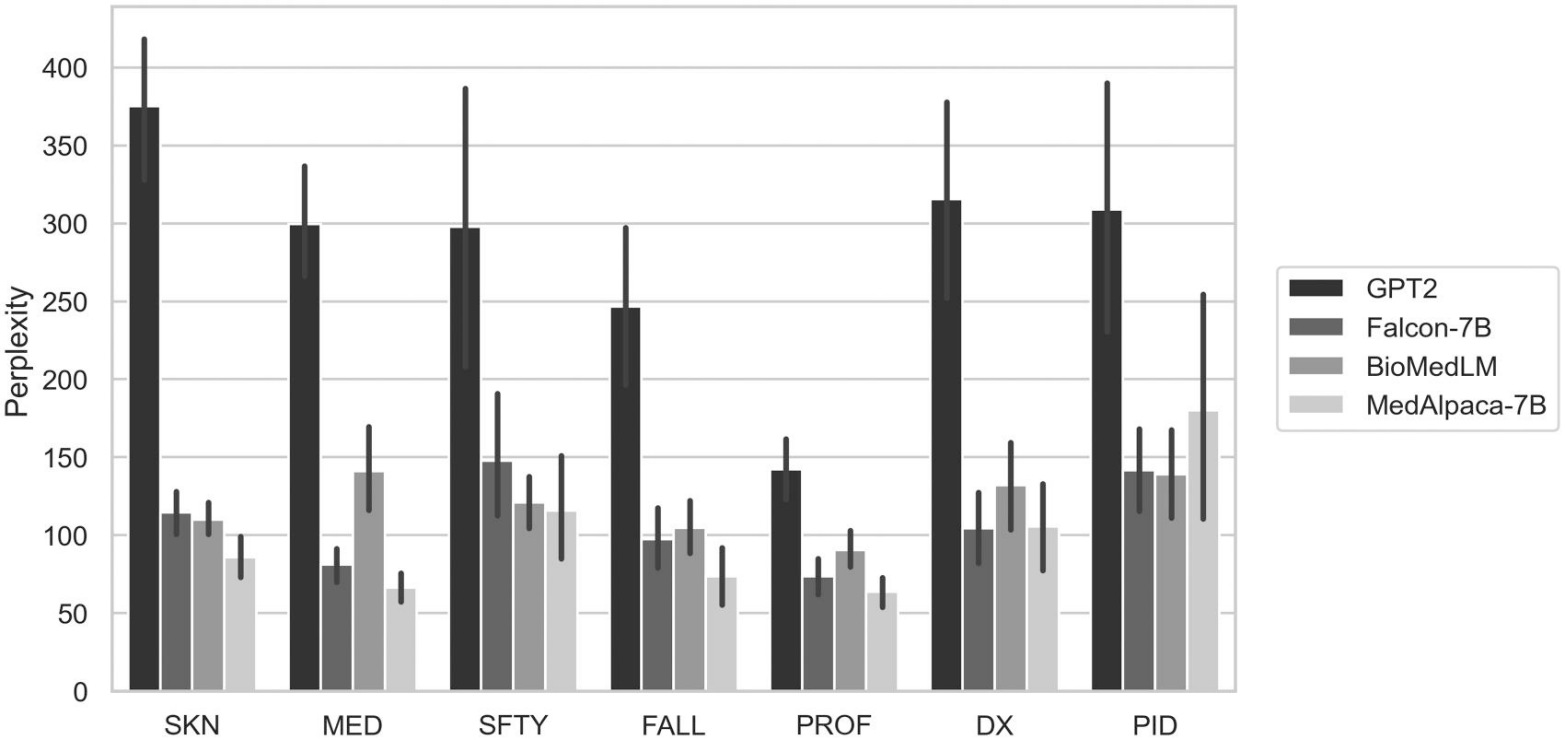
Mean perplexity with standard error bars for GPT2, Falcon-7B, BioMedLM, and MedAlpaca-7B across seven event categories: skin and tissue (SKN), medication and fluid (MED), staff safety and security (SFTY), fall (FALL), professional conduct (PROF), diagnosis in treatment (DX), and patient identification and documentation (PID).

LLMs had statistically significant different perplexity measures for skin and tissue, medication and fluid, fall, and professional conduct event categories (*P* < .001), summarized in Table 2 and Figure 2. This difference was driven by GPT2’s overall poor performance (high perplexity) and MedAlpaca-7B’s good performance (low perplexity). Falcon-7B performed slightly better than BioMedLM for medication and fluid, fall, and professional conduct events. The significant differences in diagnosis in treatment events (*P* = .001) was again driven by GPT2’s poor performance. Falcon-7B performed slightly better than MedAlpaca-7B with an average perplexity of 104.5 and 105.6, respectively. For staff safety and security events (*P* = .037), MedAlpaca-7B again performed the best, followed by BioMedLM and Falcon-7B. There was no statistically significant difference in perplexity measures for patient identification and documentation (*P* = .153). For this event, category BioMedLM had the best performance followed by Falcon-7B, with mean perplexities of 138.9 and 141.6, respectively. Additionally, target sentences with the lowest average perplexities for each event category are shown in Table 3 with the target sentences’ target token. The target tokens in the sentences with the lowest perplexity tended to be for skin (shortness, defined), fall (conservation, exercises), and medication (trimethoprim, copay) event categories. An expanded list of the top one hundred lowest perplexity target tokens across event categories is included in Supplementary Appendix A.

**Table 3.** Target tokens and sentences with the lowest average perplexities for each event category.

Target token	Event category	Average perplexity	Target sentence
Shortness	SKN	11.9	Denied any fever, chills, chest pain, **shortness** of breath, abdominal pain, nausea/vomiting…
Conservation	FALL	12.9	Extensive review of energy **conservation** strategies during functional tasks…
Trimethoprim	MED	15.2	If a carbapenem is initiated and susceptibility to ciprofloxacin, levofloxacin, or **trimethoprim**-sulfamethoxazole is demonstrated…
Exercises	FALL	15.3	She was able to ambulate with rollator and completed **exercises** and functional activities training…
Copay	MED	16.2	Patient was discharged on two medications, but was only able to get one of the medications due to insurance **copay**.
Boyfriend	SFTY	16.6	Female was intoxicated sitting in a wheel chair talking to paramedic crying and talking about her **boyfriend** and being very aggressive towards hospital staff verbally…
Defined	SKN	17.9	chest x-ray showed poorly **defined** alveolar infiltrate in the right upper lobe
Ignore	PROF	17.9	She angrily entered the nurse's station where I was performing documentation, and belligerently said to me “Are you going to do something about the pump beeping or are you just going to **ignore** it?”
Role	PROF	19.4	We know everyone has a **role** to play in a patient's care, I recommend that we all be cordial and respectful of each other.
Threats	SFTY	21.6	Patient then proceeded to spit in my face and laugh, making **threats** and stating my name out loud and saying “I know your face and your name and I'm coming back here to get you”
Untreated	DX	22.0	The patient is admitted with Staph aureus bacteremia and vertebral osteomyelitis abscess, probably as a result of **untreated** Staph.
Spelled	PID	27.3	discovery that the patient's first name was **spelled** incorrectly in the chart which is why the charts were not linked
Armband	PID	32.0	I asked the patient their date of birth, but I discovered that it did not match her information on his ID **armband**, stickers, or computer.
Tangent	DX	36.3	Treatment Fields: 1 Medial **Tangent** RT Breast 6X…

The target token in the target sentence is bolded.

Clinical models, particularly MedAlpaca-7B, tended to perform better on sentences with clinical narratives, Table 4. Falcon-7B tended to perform better on sentences with more colloquial language and discussion topics about communication (eg, “to cooperate,” “I called,” “misunderstanding”). Clinical and general LLMs had similar performance when sentences discussed clinical workflow topics (eg, “did not match,” “patient care”). Interestingly, Falcon-7B tended to perform better for lists even when the lists are of medical concepts. However, this could be an artifact of subsequent meanings of clinical shorthand.

**Table 4.** Examples of target sentences from PSE reports in which clinical LLM (BioMedLM and MedAlpaca-7B) perplexity measures were lower, higher, and comparable to general LLMs (GPT2 and Falcon-7B), top, middle, and bottom, respectively.

Examples of target sentences from PSE reports	Comparison of perplexity performance
Patient was on inhaled epoprostenol and being weaned off. (Medication and fluid) Pt was placed on a diet despite presence of slurred speech [supported by documented nih score] and documented failed BSS. (Diagnosis in treatment) Patient had a fall on Wednesday morning. (Fall)	• Better performance (lower perplexity) with clinical LLMs
I then call staff to clear what continues to appear to be a misunderstanding of the situation. (Professional conduct) Security was called and assisted in getting the patient to cooperate as they helped escort him off the property due to his behavior. (Staff safety and security) 1 Medial Tangent LT Breast 10X: Treated 1A Medial Tangent LT Breast 6X Partially Treated: Patient received 31.5 of 51.2 MU's 2 Lat Tangent LT Breast 15X: Fraction 11 of 16. (Diagnosis in treatment)	• Better performance (lower perplexity) with general LLMs
I asked the patient their date of birth, but I discovered that it did not match her information on her ID armband, stickers, or computer. (Patient identification and documentation) We know everyone has a role to play in a patient's care (Professional conduct)	• Similar performance (perplexity)

The associated event category for the sentence is indicated in parentheses.

Although MedAlpaca-7B and Falcon-7B had overall better performance, they also had the longest running time largely due to their model and parameter sizes. The total number of parameters of a model determines the total number of computations and memory requirements, impacting performance. MedAlpaca-7B and Falcon-7B each have nearly 55 times and 2.5 times as many parameters as GPT2 and BioMedLM, respectively. On a 16GB RAM CPU, Falcon-7B completed the 600-sentence perplexity task in 265 min, MedAlpaca-7B in 220 min, BioMedLM in 63 min, and GPT2 in 5 min.

## Discussion

LLMs offer tremendous potential to transform the process of analyzing PSE reports to identify key insights that can improve patient safety. However, for LLMs to support PSE report analysis there must be a good fit between the language model and the nature of the text used in safety event reports. Our results demonstrate variability in the perplexity scores across tokens and models. This variability in perplexity scores could be suggestive of several factors including the complexity of the language in the target sentences, the specificity of medical terminology, and how well each model handles certain types of tokens based on its pre-training capabilities. For example, “[shortness] of breath” and “[conversation] of energy” are phrases that could appear in both general and clinical corpora with similar context, leading to lower average perplexities across the models. On the other hand, terms such as “clocked,” “job,” and “impatient” had higher average perplexity in the context of professional conduct. While these terms are common in general language, they could take on more nuanced meaning in healthcare settings when relating to staff conduct. Useful LLMs should add value to identify insights from safety reports and reduce the work burdens a patient safety officer might feel without use of the LLMs. We developed an easy-to-use approach to assess LLMs in the context of PSE report analysis that can be used by healthcare facilities, patient safety organizations, federal agencies, and other stakeholders that collect and analyze safety event reports.

### Leveraging for patient safety

Although our results show that there is no clear winner and that a single LLM approach may not be the most useful strategy for analyzing PSE reports, our assessment results have several implications for stakeholders that are seeking to leverage LLMs. Parameter size tended to be more important than vocabulary size. Falcon7B and MedAlpaca7B had the largest parameter sizes and generally performed better than their smaller counterparts. This reflects the general benefits of model size, namely an increased capacity for learning more complex relationships between words and concepts and better generalization across content types. GPT2, on the other hand, had the second largest vocabulary but vastly fewer parameters and was the worst performer of the 4 models. Although MedAlpaca-7B tended to perform better for most event categories, MedAlpaca-7B took much longer to run, which might make it impractical for healthcare systems without sufficient parallel processing capabilities. Clinical language models (BioMedLM and MedAlpaca-7B) tended to perform better for sentences with clinical narratives, often reported by nurses and physicians. General models (Falcon7B and GPT2) had better performance for sentences about professional conduct and communication themes as these sentences tend to have a more diverse range of reporters and are often not as clinically focused. Lastly, our findings also suggest that smaller models, such as BioMedLM, can have comparable performance to models twice its size when using custom tokenizers. Leveraging smaller models and custom tokenizers to achieve better encoding of medically-specific terminology may be a useful lower compute option for healthcare systems. Given these results, leveraging multiple LLMs may be the most optimal approach with specific LLMs for certain event type categories.

### Implementing in healthcare systems

Importantly, our approach provides a process by which healthcare facilities, and other stakeholders, can assess LLMs to meet their unique needs. Facilities can create tokens and target sentences based on their datasets to evaluate LLMs. To do this requires data scientists working in partnership with clinical experts that can identify relevant tokens and sentences from a patient safety perspective. This evaluation can be routinely conducted, for example, on a quarterly basis, using the latest PSE data to assess any changes in previously used or newer LLM's goodness of fit. This routine assessment helps ensure that the models remain aligned with the evolving nature of PSE data. There is also the opportunity to embed LLMs into existing PSE reporting software systems. This will require advancements to these systems so that technical integration with LLMs is feasible and that user interfaces are appropriately designed to support both reporters of safety issues and the patient safety officers that are charged with analyzing these data.

### Limitations

Perplexity is one of many metrics to evaluate LLMs and perplexity alone does not comprehensively capture the model's performance for specific tasks. A preliminary analysis of the BERTScore F1 metric suggests that while BERTScore often aligns with perplexity (Supplementary Appendix B), a more comprehensive evaluation would be beneficial.^21^ The PSE reports originate from one healthcare system. Although multiple hospitals and healthcare settings are represented in this one healthcare system, expanding the analysis to include more healthcare systems would enhance the generalizability of our methods and findings. In addition, we were not able to evaluate bias and fairness of the LLMs due to the lack of demographic information related to patients, reporters, or medical staff involved.

Overall, our results also highlight the need for improvements in LLM performance when applied to PSE report text. Part of the challenge for LLMs may be the lack of publicly available patient safety data for training development and training of the model. Although there are some public safety data available, primarily through the FDA, these databases are narrowly focused on equipment, devices, medications, and vaccine events which are a small fraction of PSE report topics. Improving LLM performance for safety will require fine-tuning and augmentation techniques to effectively handle more patient safety domain specific language. This may require access to large databases of PSE reports and should be explored in future work.

## Conclusion

Healthcare facilities and other stakeholders are often challenged with how to effectively and efficiently analyze PSE reports. Our approach provides a method that can enable healthcare facilities to rapidly assess the fit of LLMs to their PSE reports allowing them to select a set of LLMs to facilitate PSE analysis.

## Supplementary Material

ooae128_Supplementary_Data

## Data Availability

The data underlying this article cannot be shared publicly due to the sensitive and protected policies for patient safety event reports. The data will be shared on reasonable request to the corresponding author.
