# Supplementary material for: Does one size fit all? Developing an evaluation strategy to assess large language models for patient safety event report analysis
Source: JAMIA Open. 2024 Nov 9;7(4):ooae128. doi: 10.1093/jamiaopen/ooae128 (PMC11549957; doi:10.1093/jamiaopen/ooae128)
Supplement: ooae128_Supplementary_Data [file ooae128_supplementary_data.zip › Appendix A.docx]

Appendix A: Selected 100 target tokens with the lowest average perplexity across event categories.

| **Target Token** | **Event Category** | **Average Perplexity** |
| --- | --- | --- |
| shortness | skin | 11.9 |
| conservation | fall | 12.9 |
| trimethoprim | med | 15.2 |
| exercises | fall | 15.3 |
| copay | med | 16.2 |
| inhalation | med | 16.5 |
| boyfriend | safety | 16.6 |
| apartment | fall | 17.6 |
| defined | skin | 17.9 |
| ignore | prof | 17.9 |
| proteus | skin | 18.1 |
| nonskid | fall | 18.5 |
| naloxone | med | 18.6 |
| footwear | fall | 19.0 |
| role | prof | 19.4 |
| behave | prof | 19.5 |
| lethargy | skin | 19.7 |
| nasty | prof | 20.2 |
| insulins | med | 20.4 |
| backward | fall | 20.5 |
| shouted | prof | 20.8 |
| distances | fall | 20.8 |
| threats | safety | 21.6 |
| squatting | fall | 21.7 |
| descended | fall | 21.9 |
| untreated | diag | 22.0 |
| cough | skin | 22.6 |
| descent | fall | 23.2 |
| processed | med | 23.4 |
| congestive | skin | 23.4 |
| footage | safety | 23.6 |
| interrupted | prof | 23.7 |
| amoxicillin | med | 24.1 |
| landed | fall | 24.3 |
| handbook | fall | 24.6 |
| degenerative | skin | 24.7 |
| deescalate | safety | 24.8 |
| uneven | fall | 25.1 |
| unprofessional | prof | 25.5 |
| dizzy | fall | 26.0 |
| summoned | safety | 26.0 |
| pointcare | fall | 26.2 |
| walker | fall | 26.5 |
| empiric | skin | 27.0 |
| spelled | pid | 27.3 |
| punched | safety | 28.2 |
| knees | fall | 28.3 |
| argue | prof | 28.7 |
| pepcid | med | 29.1 |
| bedridden | skin | 29.4 |
| listening | prof | 29.7 |
| transaminitis | skin | 29.9 |
| wallet | safety | 30.2 |
| overridden | med | 30.3 |
| slipped | fall | 30.4 |
| hctz | med | 30.7 |
| lvef | skin | 31.0 |
| armband | pid | 32.0 |
| sounding | fall | 32.8 |
| disruptive | safety | 33.0 |
| booking | safety | 33.0 |
| gastritis | skin | 33.1 |
| boss | prof | 33.3 |
| disrespected | prof | 33.4 |
| rsv | skin | 33.7 |
| commode | fall | 33.7 |
| sideways | fall | 33.9 |
| sir | prof | 34.0 |
| gentamicin | med | 34.1 |
| absolutely | prof | 34.3 |
| intestinal | skin | 34.5 |
| professional | prof | 34.5 |
| scoot | fall | 34.5 |
| spc | fall | 34.7 |
| creatinine | skin | 34.9 |
| unprofessionalism | prof | 35.1 |
| bullying | prof | 35.1 |
| cg | fall | 35.2 |
| property | safety | 35.3 |
| schizoaffective | skin | 35.6 |
| reveal | skin | 35.7 |
| choose | prof | 35.7 |
| ignored | prof | 35.9 |
| handcuffs | safety | 35.9 |
| disease | skin | 36.1 |
| bathroom | fall | 36.2 |
| unwarranted | prof | 36.2 |
| parkinson | skin | 36.2 |
| tangent | diag | 36.3 |
| empi | pid | 36.3 |
| soap | skin | 36.6 |
| pressure | skin | 36.6 |
| dispensed | med | 36.6 |
| typo | med | 36.8 |
| crazy | prof | 37.0 |
| sacral | skin | 37.2 |
| impatient | prof | 37.3 |
| job | prof | 37.3 |
| occurence | fall | 37.6 |
| clocked | prof | 37.9 |
