# Supplementary material for: Does one size fit all? Developing an evaluation strategy to assess large language models for patient safety event report analysis
Source: JAMIA Open. 2024 Nov 9;7(4):ooae128. doi: 10.1093/jamiaopen/ooae128 (PMC11549957; doi:10.1093/jamiaopen/ooae128)
Supplement: ooae128_Supplementary_Data [file ooae128_supplementary_data.zip › Appendix B.docx]

Appendix B:

Figure S1: Preliminary analysis of BERTScore F1 metric for GPT2, Falcon-7B, BioMedLM, and MedAlpaca-7B across seven event categories: skin and tissue (SKN), medication and fluid (MED), staff safety and security (SFTY), fall (FALL), professional conduct (PROF), diagnosis in treatment (DX), and patient identification and documentation (PID).
