# Supplementary figures and images for: Does one size fit all? Developing an evaluation strategy to assess large language models for patient safety event report analysis
Source: JAMIA Open. 2024 Nov 9;7(4):ooae128. doi: 10.1093/jamiaopen/ooae128 (PMC11549957; doi:10.1093/jamiaopen/ooae128)

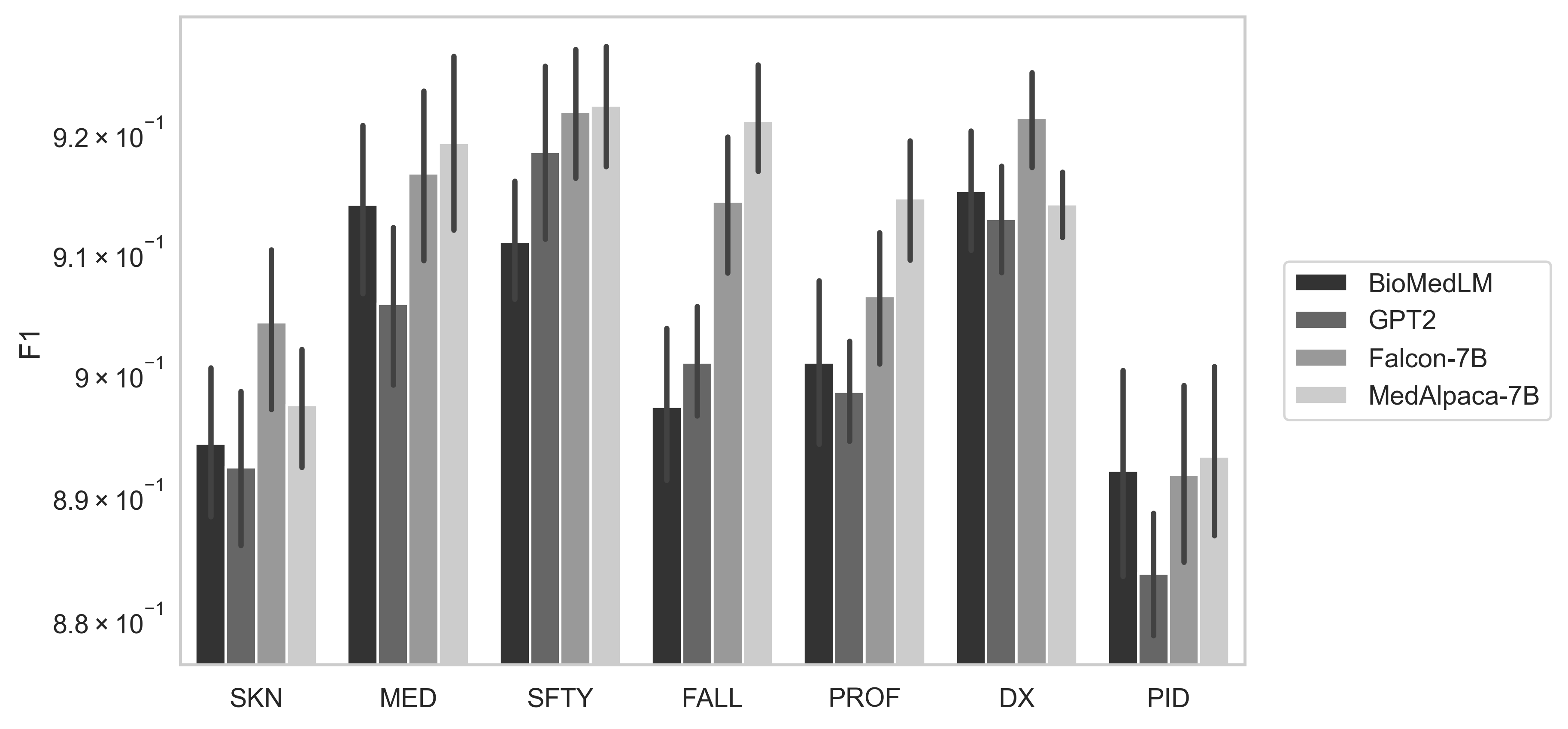

Supplement: ooae128_Supplementary_Data [file ooae128_supplementary_data.zip › BERTScore F1.png]
